# Imaging Characteristics of Malignant Sinonasal Tumors

**DOI:** 10.3390/jcm6120116

**Published:** 2017-12-06

**Authors:** Masaya Kawaguchi, Hiroki Kato, Hiroyuki Tomita, Keisuke Mizuta, Mitsuhiro Aoki, Akira Hara, Masayuki Matsuo

**Affiliations:** 1 Department of Radiology, Gifu University School of Medicine, 1-1 Yanagido, Gifu 501-1194, Japan; kawamasaya0713@yahoo.co.jp (M.K.); matsuo_m@gifu-u.ac.jp (M.M.); 2 Department of Tumor Pathology, Gifu University School of Medicine, Gifu 501-1194, Japan; ahara@gifu-u.ac.jp; 3 Department of Otolaryngology, Gifu University School of Medicine, Gifu 501-1194, Japan; kmizuta@gifu-u.ac.jp (K.M.); aoki@gifu-u.ac.jp (M.A.)

**Keywords:** sinonasal tract, malignant tumor, CT, MRI

## Abstract

Malignancies of the nasal cavity and paranasal sinuses account for 1% of all malignancies and 3% of malignancies of the upper aerodigestive tract. In the sinonasal tract, nearly half of all malignancies arise in the nasal cavity, whereas most of the remaining malignancies arise in the maxillary or ethmoid sinus. Squamous cell carcinoma is the most common histological subtype of malignant tumors occurring in this area, followed by other epithelial carcinomas, lymphomas, and malignant soft tissue tumors. Although many of these tumors present with nonspecific symptoms, each tumor exhibits characteristic imaging features. Although complex anatomy and various normal variants of the sinonasal tract cause difficulty in identifying the origin and extension of large sinonasal tumors, the invasion of vital structures such as the brain, optic nerves, and internal carotid artery affects patients’ prognosis. Thus, diagnostic imaging plays a key role in predicting the histological subtype and in evaluating a tumor extension into adjacent structures. This article describes the computed tomography and magnetic resonance imaging findings for malignant sinonasal tumors.

## 1. Introduction

Sinonasal neoplasms are relatively rare and malignant sinonasal neoplasms are more common than their benign counterparts. Sinonasal malignancies comprise only 3% of all head and neck cancers and 1% of all malignancies [1,2,3]. The complex anatomy of the region and the rare occurrence of these tumors pose diagnostic and therapeutic challenges. Of the various histological subtypes of malignant sinonasal tumors, squamous cell carcinoma (SCC) is the most common subtype, whereas the other subtypes, such as adenocarcinoma, minor salivary gland carcinoma, undifferentiated carcinoma, neuroendocrine carcinoma, and nonepithelial malignancies (such as sarcoma, lymphoma, plasmacytoma, olfactory neuroblastoma, and melanoma) are considerably less common. The treatment modalities vary depending on the tumor histological subtype, location, and extent of the disease and include surgery, radiation, chemotherapy, or a combination of two or more of these modalities. The prognosis of the patients largely depends on tumor histology, location, and stage.

## 2. Anatomy

The sinonasal tract comprises the nasal cavity, maxillary sinus, ethmoid sinus, frontal sinus, and sphenoid sinus. It includes various tissue types such as epithelium, mucosal epithelium, vessel, nerve, cartilage, bone, and lymphatic tissue. The maxillary, ethmoid, nasal, frontal, palatine, sphenoid, and lacrimal bones are also included. This area comprises bone and cartilage lined with ciliated respiratory epithelium and is located between the orbit and the oral cavity. This area is also close to the frontal cortex through the cribriform plate of the ethmoid bone and is connected with the cerebrum by vessels, lymph channels, and nerves.

The nasal cavity is divided by the nasal septum in the midline. Bilateral nasal cavities include the superior, middle, and inferior turbinates; in addition, the nasal meatus is located under each of them. The common nasal meatus lies between the nasal turbinate and the nasal septum, and the olfactory cleft is located superior to the lower border of the middle turbinate. The paranasal sinuses are connected to the nasal cavity and categorized according to the location of the ostium. The middle meatus drains the maxillary, frontal, and anterior ethmoid sinuses, whereas the superior meatus drains the posterior ethmoid and sphenoidal sinuses. These are located close to important structures, such as the cavernous sinus, the internal carotid artery, the pituitary gland, and the optic nerve.

The upper third of the nasal cavity, the frontal sinus, and parts of the ethmoid and sphenoid sinuses are supplied by the ophthalmic artery, whereas most of the remaining sinonasal tracts are supplied by facial and maxillary arteries. The olfactory mucosa in the upper part of the nasal cavity is innervated by the olfactory nerve. The ophthalmic nerve (V1) provides sensory innervation to the ethmoid sinus, sphenoid sinus, lateral wall of the nasal cavity, and the anterior part of the nasal septum. The maxillary nerve (V2) provides sensory innervation to the maxillary sinus. Submandibular lymph nodes drain the anterior components of the sinonasal tract (anterior drainage pathway), whereas the retropharyngeal lymph nodes drain the posterior components of the sinonasal tract (posterior drainage pathway). These routes of lymphatic drainage finally reach the superior deep cervical lymph nodes.

## 3. Clinical Presentation

The clinical presentations of sinonasal malignancies are nonspecific and identical to those of inflammatory sinus disease, such as nasal obstruction, rhinorrhea, epistaxis, headache, and facial pain. In addition, these malignancies are often asymptomatic until they erode and invade the adjacent structures. Therefore, sinonasal malignancies are usually diagnosed in advanced stages, and the survival rate and prognosis of these patients remain poor. Sinonasal malignancies should be considered in patients with unilateral nasal obstruction or recurrent epistaxis. Progressive sinonasal malignancies that often invade the adjacent structures produce characteristic symptoms, such as cranial neuropathies due to intracranial extension, facial subcutaneous soft tissue swelling, exophthalmos, diplopia, visual disturbances, eye movement disorders, olfactory dysfunction, and respiratory symptoms. Early detection and adequate treatment are required to improve the survival and mortality rates.

## 4. Imaging

Computed tomography (CT) and magnetic resonance imaging (MRI) are very useful tools for the assessment of tumor size, nature, extent, and invasion. The evaluation of the potential extension into adjacent regions impacts the surgical or therapeutic planning, particularly in cases with the involvement of the anterior and middle cranial fossa, orbit, pterygopalatine fossa, palate, or infratemporal fossa (masticator and parapharyngeal space). CT is the most commonly used imaging modality because of its wider availability, easy access, lower cost, and potential to offer greater anatomic detail. In comparison to MRI, CT is particularly effective in delineating calcification and evaluating the pattern of bone invasion. Intralesional calcifications are observed in some sinonasal disorders, such as adenocarcinoma, olfactory neuroblastoma, inverted papilloma, fibrous dysplasia, osteoma, osteosarcoma, cartilaginous tumor, fungal sinusitis, and dentigerous tumor. Characteristic patterns of bone invasion help predict the tumor histology. High grade malignancies show extensive bony destruction, whereas small round cell tumors show permeative invasion and lack of bony destruction. Benign lesions and low grade malignancies may cause bony expansion due to their slow and expansile growth. Contrast-enhanced CT is invaluable for the identification of the feeding artery (because of its high spatial resolution) and for the diagnosis of hypervascular tumors. In contrast, MRI provides higher contrast resolution and affords an excellent characterization of the soft tissue components of the tumor. The signal intensity within a tumor varies according to the tissue components. Malignant tumors usually exhibit nonspecific hyperintensity on T2-weighted images (T2WI) and hypo- to isointensity on T1-weighted images (T1WI). On T2WI, mucinous or cartilaginous tumors show marked hyperintensity, hypercellular tumors show slight hyperintensity, and tumors with fibrosis, calcification, or flow void show hypointensity. On T1WI, hyperintensity within a tumor is indicative of the presence of methemoglobin, melanin, lipid, protein, and mineral elements. Diffusion-weighted image (DWI) with measurement of apparent diffusion coefficient (ADC) captures the degree of Brownian movement of the water molecules in tissues, which serves as a useful imaging biomarker. Low-ADC lesions with strong diffusion restriction indicate hypercellularity, abscess, or hemorrhage, whereas high-ADC lesions indicate hypocellularity, mucus, cartilage, or fluid. Therefore, DWI with ADC measurement is usually useful to differentiate between benign and malignant tumors. The ADC values of malignant sinonasal tumors (0.87–1.10 × 10^−3^ mm^2^/s) have been shown to be significantly lower than those of benign sinonasal lesions (1.35–1.78 × 10^−3^ mm^2^/s) [4,5,6]. Contrast-enhanced MRI is a useful method in detecting perineural spread and dural invasion. Perineural spread can be diagnosed as nerve thickening, widening of the neural foramen, loss of perineural fat, and enhancement of the nerve. Linear enhancement alone is not a conclusive sign of dural invasion; however, dural thickening > 5 mm, pial enhancement, or the presence of focal dural nodules are indicative of dural invasion.

## 5. Sinonasal Malignancies

### 5.1. Squamous Cell Carcinoma

Squamous cell carcinoma (SCC) is the most common histological subtype and accounts for more than half of all sinonasal malignant tumors. It most commonly affects patients in the sixth and seventh decade of life with male predominance. The maxillary sinus is the most frequently affected site, followed by the nasal cavity and the ethmoid sinus. Primary SCCs of the sphenoid sinus and frontal sinus are rare. In the past, sinonasal SCC has been linked to cigarette smoking and occupational exposures [7]. However, in recent years, the generational changes in sexual behavior may have led to an increased positivity rate for human papillomavirus (HPV) among patients with sinonasal SCC; consequently, HPV has been identified in 32–62% of all sinonasal SCCs [8,9]. HPV positivity is more common in SCCs of the nasal cavity than in paranasal SCCs [9]. As with oropharyngeal SCCs, patients with a HPV-positive sinonasal SCC have a better prognosis than patients with a HPV-negative sinonasal SCC [8,9].

**Figure 1:**
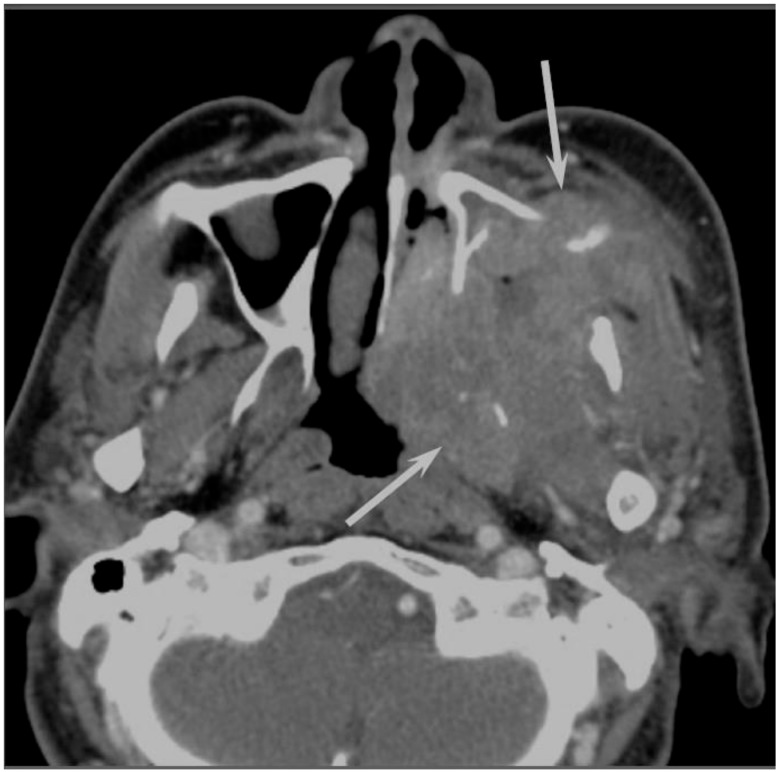
Squamous cell carcinoma of the left maxillary sinus. Contrast-enhanced CT image showing an ill-demarcated, heterogeneously enhanced bulky mass with extensive bony destruction (arrows).

Sinonasal SCCs are characterized by aggressive bony destruction of the adjacent sinus walls (Figure 1). Because sinonasal SCCs are often detected at an advanced stage, the invasion of the contralateral sinonasal area, orbital wall, infratemporal fossa, and skull base is sometimes observed. Hypoxia is a common feature in most cases of SCC, and prolonged oxygen deprivation often leads to chronic hypoxic stress and consequent tumor necrosis [10]. Thus, intratumoral necrosis is also one of the characteristic findings in SCCs. On MRI, isointensity on T1WI, slight hyperintensity on T2WI, and moderate enhancement on contrast-enhanced T1WI are typical and nonspecific imaging findings for SCCs. Smaller lesions are typically homogeneous, whereas larger tumors are usually more heterogeneous and exhibit areas of necrosis and hemorrhage [11]. In the maxillary sinus, the ADC values of SCC (0.95 × 10^−3^ mm^2^/s) were higher than those of non-Hodgkin’s lymphoma (NHL) (0.61 × 10^−3^ mm^2^/s) [12].

### 5.2. Adenocarcinoma

Adenocarcinomas account for 10–20% of all sinonasal malignancies. Sinonasal adenocarcinomas are categorized into salivary type adenocarcinomas and non-salivary type adenocarcinomas [13]. The latter are further classified into intestinal-type adenocarcinomas (ITAC) and nonintestinal-type adenocarcinomas (non-ITAC) [13]. Sinonasal ITACs, which histopathologically resemble colorectal adenocarcinoma, can occur sporadically or are associated with occupational exposure to hardwood and leather dust. Sinonasal non-ITACs do not exhibit the histopathological features of sinonasal ITACs or of salivary type adenocarcinomas; they are categorized into high-grade type and low-grade type. Most sinonasal non-ITACs are of the low-grade type, whereas high-grade non-ITACs are rare. Although the age of the patients with sinonasal ITAC and non-ITAC may vary widely, patients in the sixth decade of life are most commonly affected. The nasal cavity is the most common site for ITAC and non-ITAC, whereas the paranasal sinuses are less commonly affected [14]. 

**Figure 2:**
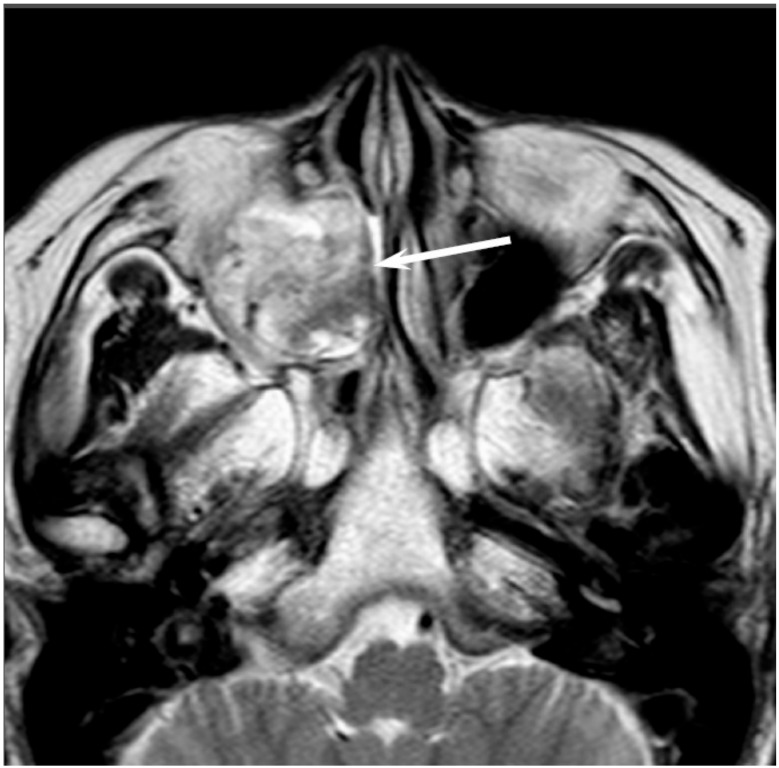
Non-intestinal type adenocarcinomas of the right maxillary sinus. T2-weighted image showing a heterogeneously hyperintense lesion (arrow).

On CT, sinonasal adenocarcinomas appear as a soft-tissue mass and occasionally exhibit areas of calcification, which reflect the mucin content. In unilateral olfactory cleft adenocarcinomas, the bulging of the nasal septum across the midline and widening of the olfactory cleft are observed [15]. High-grade adenocarcinomas often show bone destruction. Adenocarcinomas arising from the ethmoid sinus may potentially extend to the skull base and intracranially to the frontal lobes [16]. On MRI, the signal intensity of the adenocarcinomas varies according to their mucin content, cellularity and the presence of hemorrhage. Mucin-producing adenocarcinomas usually show hyperintensity on T2WI and exhibit gradual enhancement on contrast-enhanced T1WI, whereas adenocarcinomas without mucin production show iso- to hypointensity on T2WI. The imaging characteristics of adenocarcinomas are often indistinguishable from those of SCCs (Figure 2).

### 5.3. Adenoid Cystic Carcinoma

Adenoid cystic carcinomaa (ACC) are slow-growing and relentless salivary gland tumors comprising epithelial and myoepithelial neoplastic cells. ACCs are the most common malignant salivary gland tumors of the sinonasal tract; sinonasal ACCs account for 10–25% of all head and neck ACCs. The average age at presentation is the fifth to sixth decade of life. The maxillary sinus is the most commonly affected primary site, followed by the nasal cavity, ethmoid sinus, and sphenoid sinus [17]. There are three distinct histopathological subtypes of ACC: tubular, cribriform, and solid subtype. ACCs are characterized by wide local infiltration, perineural spread, a propensity for local recurrence, and late distant metastasis. Bone invasion (41%), perineural invasion (40%), and angioinvasion (3.8%) are observed in the surgical specimens of sinonasal ACCs [17]. Lymph node and distant metastases are uncommon at presentation, but the reported overall recurrence rate is 56.2% [17]. The most common sites of distant metastases are the lungs, followed by the liver and bone.

**Figure 3:**
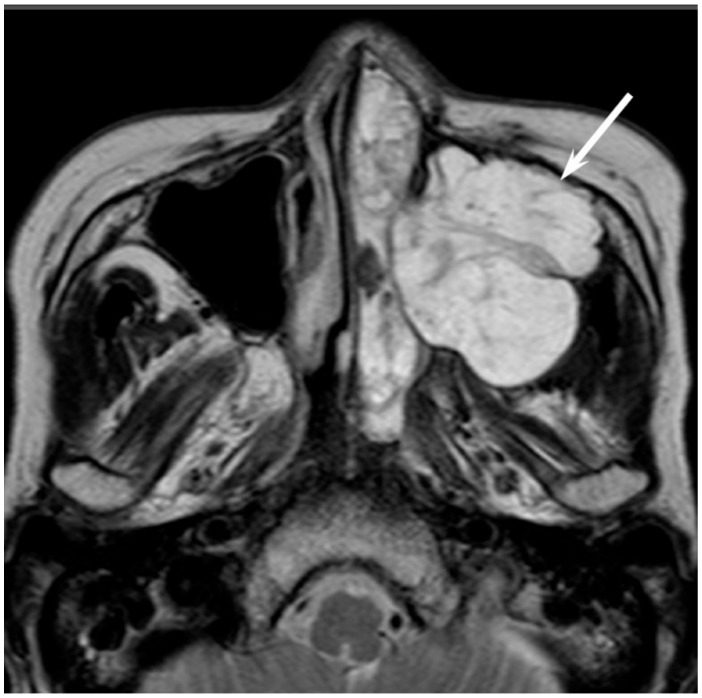
Adenoid cystic carcinoma of the left maxillary sinus and nasal cavity. T2-weighted image showing a well-demarcated, lobulated, heterogeneously and strongly hyperintense lesion (arrow).

Low-grade sinonasal ACCs may present as polypoid lesions that remodel the bone and mimic a simple polyp, whereas high-grade sinonasal ACCs may present as large irregular masses with bone destruction and heterogeneous density or signal intensity [18]. The growth pattern of maxillary sinus ACCs can be classified into expansile type with minimal bony defects and destructive type with extensive bony defects, and these tumors usually extend to the nasal cavity and, occasionally, to the retroantral fat pad, pterygopalatine fossa, or orbit [19]. ACCs show isointensity on T1WI and iso- to hyperintensity on T2WI, depending on the amount of cellularity (Figure 3). ACCs exhibit the greatest propensity for perineural spread, and the maxillary division of the trigeminal nerve is most commonly affected by sinonasal ACCs. These tumors sometimes easily extend into intracranial components including the cavernous sinus and the Gasserian ganglion, which are far away from the original site [20,21]. Furthermore, for the surgeons, it is often important to first evaluate on images whether the tumor is resectable or not and far away from vital structures. In cases with an advanced tumor, fluid collection and thickened mucosa caused by the isolated sinuses sometimes make it difficult to diagnose and stage the disease.

### 5.4. Sinonasal Undifferentiated Carcinoma

Sinonasal undifferentiated carcinoma (SNUC) is a rare and highly aggressive malignancy, which accounts for approximately 3–5% of all sinonasal cancers. SNUC is a clinicopathologically distinct carcinoma of uncertain histogenesis with no glandular or squamous features. The median age at presentation is the sixth decade of life; the reported male-to-female ratio is 2–3:1. SNUC most commonly arises from the superior nasal cavity and the ethmoid sinus. SNUC usually presents as a rapidly enlarging tumor, and the majority of these patients presents with Stage IV disease. Orbital and intracranial invasion, nodal involvement, and distant metastasis are frequent findings. The recurrence rate is 42.3%. The time to recurrence ranges from 3 to 33 months; 32.1% of patients die of local disease, whereas 14.3% of patients die of metastatic disease [22].

**Figure 4:**
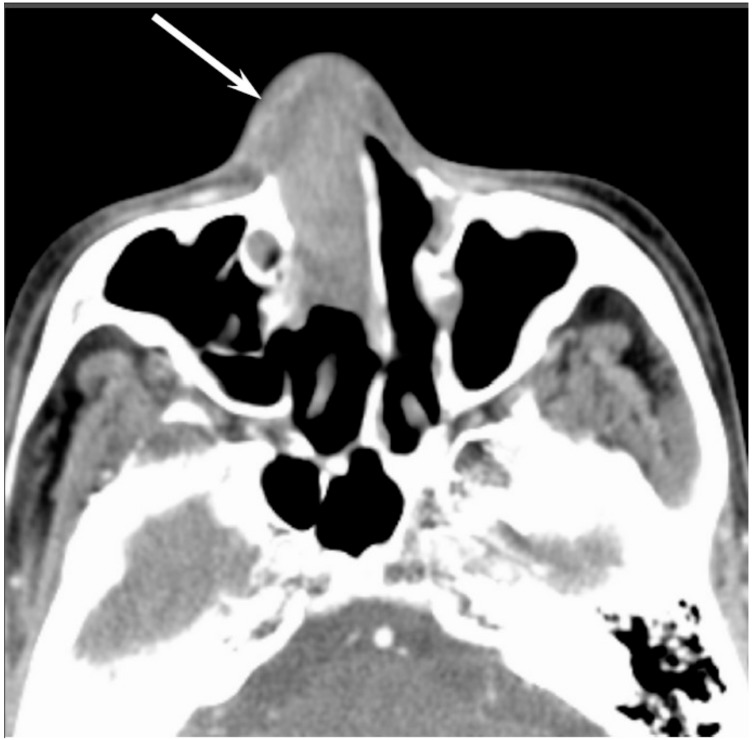
Sinonasal undifferentiated carcinoma of the right nasal cavity. Contrast-enhanced CT image showing an ill-demarcated, heterogeneously enhanced lesion (arrow).

Most SNUCs are larger than 4 cm in maximal diameter at presentation and have ill-defined margins [23]. The aggressive nature of the tumor is reflected in the bone destruction and invasion of adjacent structures, including the paranasal sinuses, anterior fossa, orbit, pterygopalatine fossa, parapharyngeal space, and cavernous sinus [23]. On CT, SNUCs usually appear as a noncalcified mass and show variable contrast enhancement and areas of central necrosis. On MRI, SNUCs show isointensity on T1WI, iso- to hyperintensity on T2WI, and exhibit heterogeneous enhancement on contrast-enhanced T1WI. Owing to the nonspecific imaging findings, it is typically difficult to distinguish between SNUCs and SCCs (Figure 4).

### 5.5. Malignant Lymphoma

The head and neck region is the second most common site for extranodal lymphomas after the gastrointestinal tract. NHL is the second most common malignancy in the sinonasal tract after SCC. Patients classically present in the sixth to eighth decades of life; the reported male-to-female ratio is 2:1. Diffuse large B-cell lymphoma (DLBCL) most commonly arises from the paranasal sinuses; the maxillary sinus is the most common site of involvement, although DLBCL may arise from the nasal cavity. NK/T-cell lymphoma most commonly involves the nasal cavity and shows a predilection for occurrence in Asian and South American populations. B-cell lymphoma has a better prognosis than T-cell lymphoma. 

**Figure 5:**
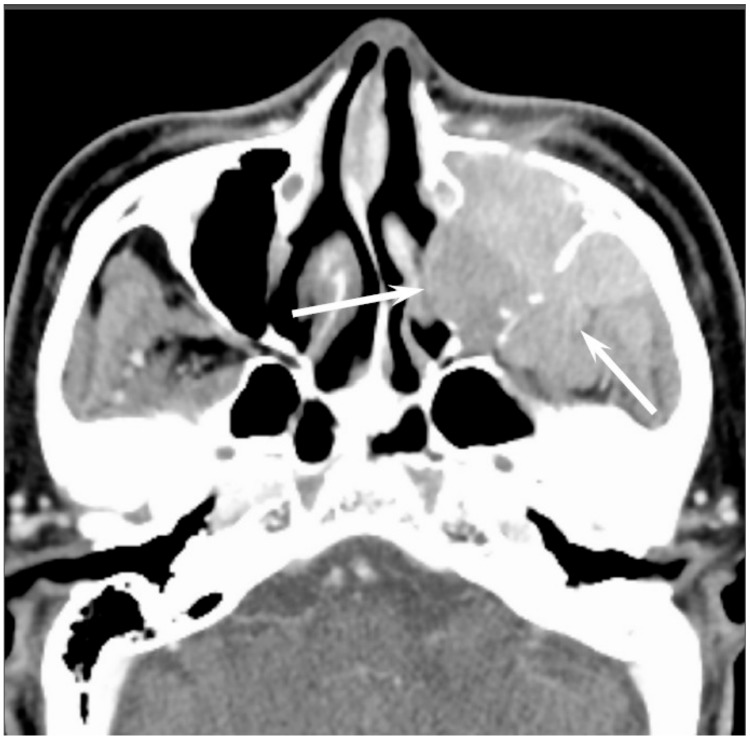
Diffuse large B-cell lymphoma of the left maxillary sinus. Contrast-enhanced CT image showing a homogeneously enhanced lesion accompanied by remaining sinus walls as a linear structure within the tumor (arrows).

On CT, sinonasal lymphomas frequently show both infiltrative or permeative bony invasion and exhibit varying degrees of regional bony destruction [12]. NHLs with permeative-type tumor invasion typically cross the sinus wall and exhibit remnants of sinus wall as a linear structure within the tumor (Figure 5) [24]. In contrast, bony resorption or remodeling caused by the lymphoma may also be accompanied by bone sclerosis [25]. NHLs usually show isointensity on T1WI and slightly hyperintensity on T2WI [11]. Although the signal intensity of NHLs is nonspecific, the ADC measurement helps differentiate these tumors from other malignancies. In the maxillary sinus, the ADC values of NHL (0.61 × 10^−3^ mm^2^/s) were shown to be lower than those of SCCs (0.95 × 10^−3^ mm^2^/s), which reflects the greater cellularity of NHLs [12]. Although NHLs usually appear as a homogeneously enhanced mass, necrotic areas within the tumor are occasionally observed in NK/T-cell lymphoma [26,27].

### 5.6. Extramedullary Plasmacytoma

Extramedullary plasmacytomas (EPMs) (also referred to as extraosseous plasmacytomas) are characterized by soft-tissue monochronal plasma cell proliferation with no evidence of underlying multiple myeloma; these tumors account for 4% of all plasma cell tumors. EPMs usually occur in the sixth decade of life; the reported male-to-female ratio is 3–4:1. Approximately 80% of EPMs involve the head and neck region; the nasal cavity and paranasal sinuses are most commonly affected, followed by the nasopharynx, oropharynx, and larynx [28]. Regional recurrence or spread to other osseous sites may occur. Approximately 15% of the patients develop multiple myeloma [28]. The prognosis depends on the tumor size (>5 cm) and nodal involvement [29].

**Figure 6:**
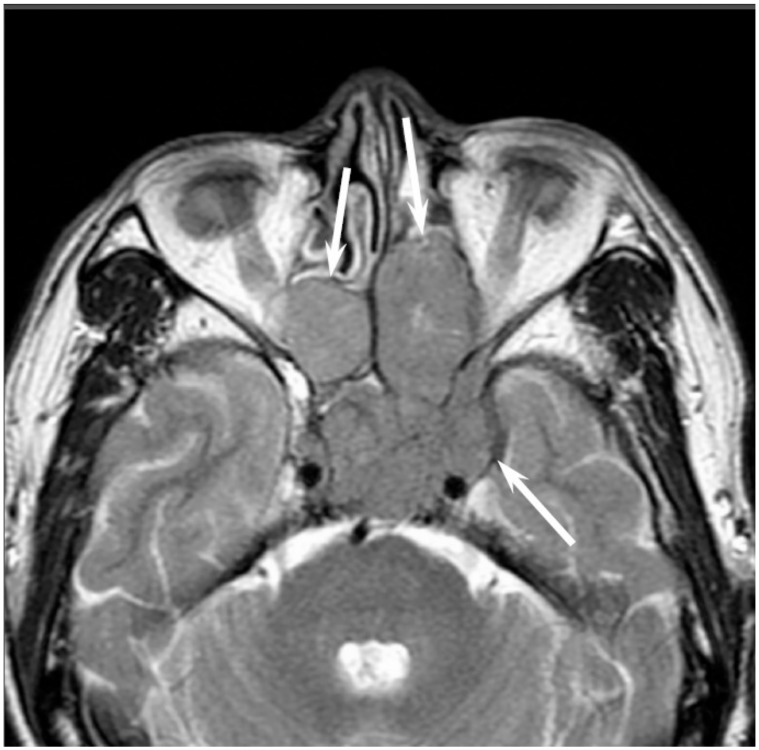
Extramedullary plasmacytoma of the bilateral nasal cavity. T2-weighted image showing a homogeneously isointense lesion (arrows).

On CT, EMPs typically appear as well-defined, polypoid soft-tissue masses, which exhibit homogenous enhancement. Large tumors may show areas of necrosis, destruction of the adjacent bone, infiltration of the adjacent structures, and vascular encasement [30,31]. On MRI, EMPs show isointensity on T1WI, iso- to slight hyperintensity on T2WI, and exhibit variable enhancement on contrast-enhanced T1WI [30,31] (Figure 6). Because they are highly vascularized tumors, vascular flow void may be observed within the tumor.

### 5.7. Olfactory Neuroblastoma

Olfactory neuroblastomas (ONB) arise from the specialized sensory neuroepithelial (neuroectodermal) olfactory cells that are normally found in the cribriform plate, the superior turbinate, and the upper third of the nasal septum. ONBs account for 3% of all sinonasal tumors [32]. ONB may occur at any age, with a peak incidence in the fifth and sixth decades of life; the reported male-to-female ratio is 1.2:1. Direct tumor extension into the adjacent paranasal sinuses, cribriform plate, skull base, orbit, and intracranial cavity is frequently observed. Cervical lymph node metastasis (20–25%) and distant metastases (10–40%) develop over the course of the disease. The most frequent sites of distant metastasis are the lungs, liver, and bone. The most commonly used staging systems are the modified Kadish and Dulguerov classifications.

**Figure 7:**
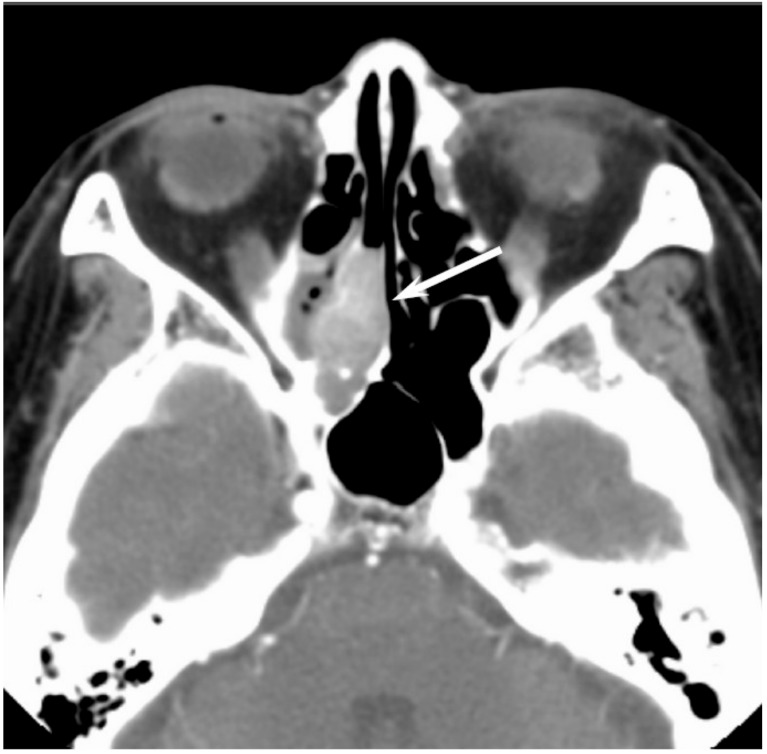
Olfactory neuroblastoma of the right nasal cavity. Contrast-enhanced CT image showing a homogeneously enhanced lesion in the right olfactory cleft (arrow).

On CT, ONBs appear as a homogeneous, well-defined soft-tissue mass. Scattered speckled calcifications may be observed within the tumor. The tumor commonly extends into the ethmoid and maxillary sinuses, but rarely involves the sphenoid sinus. CT is essential for the evaluation of osseous involvement of the cribriform plate, fovea ethmoidalis, and lamina papyracea. On MRI, ONBs usually show hypointensity relative to the gray matter on T1WI and hyperintensity relative to the gray matter on T2WI [33]. These tumors demonstrate an avid and homogeneous enhancement except for occasional areas of necrosis or hemorrhage (Figure 7). When an intracranial extension is present, the peripheral or marginal cysts are a characteristic and specific feature of ONBs [34].

### 5.8. Malignant Melanoma

Malignant melanoma (MM) originates from the pigment-producing cells (melanocytes) predominantly located in the skin. Sinonasal mucosal MMs account for 0.5–2% of all MMs and approximately 4% of all head and neck MMs. The age at occurrence may vary widely; the peak incidence is in the seventh decade of life. The tumor exhibits no gender predilection. The nasal cavity (including nasal septum, inferior and middle turbinates, and lateral nasal wall) is the second most common site for mucosal melanoma followed by the oral cavity. The paranasal sinuses are rarely affected; of these, the maxillary sinus is the most commonly affected. The occurrence of regional and distant metastases is relatively common (≥25% in most series) [35].

**Figure 8:**
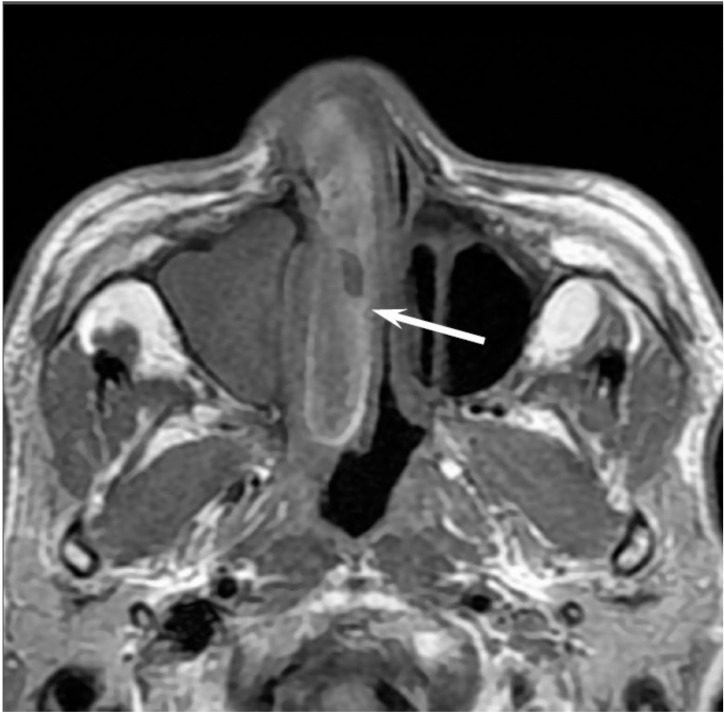
Malignant melanoma of the right nasal cavity. T1-weighted image showing heterogeneously hyperintense areas within tumor (arrow).

CT findings are nonspecific; however, CT is useful for the evaluation of the remodeling of the the surrounding bone or bony erosion [36]. On T1WI, sinonasal MMs that contain melanin or hemorrhage usually show iso- to hyperintensity relative to the gray matter; however, amelanotic melanoma may also show hypointensity (Figure 8). T1 shortening more often appears as a reflection of the paramagnetic effects associated with the products of hemorrhage rather than the presence of melanin [37]. On T2WI, sinonasal MMs typically show hyperintensity relative to the gray matter; however, melanotic melanomas may show iso- to hypointensity. MMs typically show a heterogeneous strong contrast enhancement owing to the rich vascular network.

### 5.9. Rhabdomyosarcoma

Rhabdomyosarcoma (RMS) is a malignant mesenchymal tumor with skeletal muscle differentiation and is one of the most common pediatric soft tissue sarcomas, accounting for 3–5% of all malignancies in childhood. RMS is classified into embryonal, alveolar, pleomorphic, and spindle-cell subtypes. The mean age at diagnosis is 5–6 years, and 72–81% of patients are younger than 10 years; the reported male-to-female ratio is 1.3:1 [38]. Approximately 40% of all RMSs occur in the head and neck region; the most common sites are the orbit, nasopharynx, middle ear and mastoid, and sinonasal tracts. Embryonal RMS is the most common histopathological subtype occurring in the head and neck region and in the genitourinary system and is typically associated with a favorable prognosis. 

**Figure 9:**
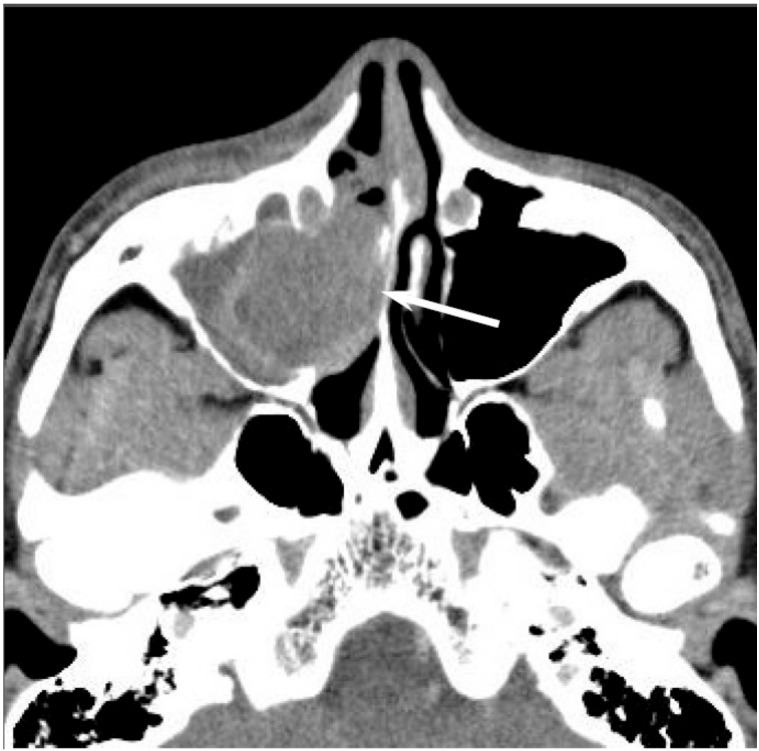
Rhabdomyosarcoma of the right nasal cavity. Contrast-enhanced CT image showing a homogeneously, slightly enhanced lesion (arrow).

The average diameter of head and neck RMSs (HNRMS) is 4.5 cm. Most of the HNRMSs have ill-defined margins with adjacent bony destruction and extension into the surrounding spaces [39,40]. On CT, HNRMSs appear as an isodense or slightly hypodense mass and show homogeneous enhancement on contrast-enhanced CT (Figure 9). Intratumoral calcification and hemorrhage rarely occur in HNRMS. On MRI, HNRMSs show isointensity relative to muscle on T1WI, and moderate hyperintensity relative to muscle on T2WI [39,40]. On contrast-enhanced T1WI, HNRMSs show various enhancement patterns; however, the majority of HNRMS shows moderate homogenous enhancement.

### 5.10. Malignant Peripheral Nerve Sheath Tumor

Malignant peripheral nerve sheath tumors (MPNST) are highly aggressive malignant mesenchymal tumors that usually arise from peripheral nerves or cells of the peripheral nerve sheath and show variable differentiation toward one of the cellular components of the nerve sheath. MPNSTs account for approximately 5–10% of all soft tissue sarcomas, of which only approximately 8–16% occur in the head and neck region [41]. They occur mainly in adults; the age at presentation may vary widely, although the peak incidence is in the fifth decade of life. They are commonly associated with neurofibromatosis type 1 (NF1) but can also arise through sporadic mutation [41]. The cases associated with NF1 occur in younger patients (mean patient age in the third to fourth decades). MPNSTs may occur in the sinonasal tract, nasopharynx, oral cavity, and orbit. Approximately two thirds of the cases metastasize, usually hematogenously, to the lung and bone.

**Figure 10:**
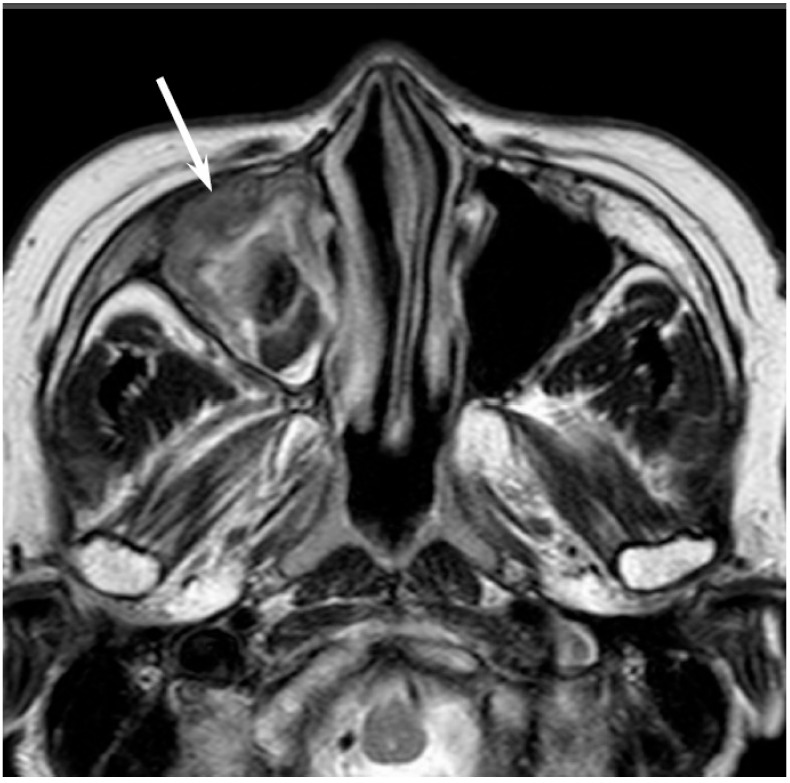
Malignant peripheral nerve sheath tumor of the right maxillary sinus. T2-weighted image showing a heterogeneously hypo- to hyperintense lesion (arrow).

On CT, MPNSTs appear as a large, hypodense soft-tissue mass with infiltration of the adjacent structures. On MRI, 51% of MPNST on T1WI and 78% of MPNST on T2WI exhibit heterogeneous signal intensity [42] (Figure 10). Compared to neurofibromas, the peripheral enhancement pattern, perilesional edema, and intratumoral cystic change are characteristic MRI findings of MPNSTs [42]. Compared to non-neurogenic malignant soft tissue tumors, the intermuscular distribution, nodular morphology with a fusiform shape, location on the course of a large nerve, and homogeneity of the signal intensity and enhancement are characteristic MRI findings of MPNSTs [43].

## 6. Conclusions

Although the radiological differentiation of sinonasal malignancies is very difficult because of the similarity of imaging findings, the tumor location, growth pattern into adjacent bone, tumor homogeneity, internal signal intensity, contrast enhancement pattern, and DWI with ADC measurement may facilitate an adequate diagnosis. CT and MRI are useful tools for pretreatment evaluation of the characterization, localization, and distribution of malignant sinonasal tumors.
